# Pharmacological Inhibition of c-Jun N-terminal Kinase Reduces Food Intake and Sensitizes Leptin’s Anorectic Signaling Actions

**DOI:** 10.1038/srep41795

**Published:** 2017-02-06

**Authors:** Su Gao, Shannon Howard, Philip V. LoGrasso

**Affiliations:** 1 Department of Molecular Therapeutics, Scripps Research Institute, Jupiter, Florida, USA

## Abstract

The role for c-Jun N-terminal Kinase (JNK) in the control of feeding and energy balance is not well understood. Here, by use of novel and highly selective JNK inhibitors, we investigated the actions of JNK in the control of feeding and body weight homeostasis. In lean mice, intraperitoneal (i.p.) or intracerebroventricular (i.c.v.) administration of SR-3306, a brain-penetrant and selective pan-JNK (JNK1/2/3) inhibitor, reduced food intake and body weight. Moreover, i.p. and i.c.v. administrations of SR11935, a brain-penetrant and JNK2/3 isoform-selective inhibitor, exerted similar anorectic effects as SR3306, which suggests JNK2 or JNK3 mediates aspect of the anorectic effect by pan-JNK inhibition. Furthermore, daily i.p. injection of SR3306 (7 days) prevented the increases in food intake and weight gain in lean mice upon high-fat diet feeding, and this injection paradigm reduced high-fat intake and obesity in diet-induced obese (DIO) mice. In the DIO mice, JNK inhibition sensitized leptin’s anorectic effect, and enhanced leptin-induced STAT3 activation in the hypothalamus. The underlying mechanisms likely involve the downregulation of SOCS3 by JNK inhibition. Collectively, our data suggest that JNK activity promotes positive energy balance, and the therapeutic intervention inhibiting JNK activities represents a promising approach to ameliorate diet-induced obesity and leptin resistance.

Body weight homeostasis is regulated by the control of the balance between energy (food) intake and energy expenditure^1^. A sustained positive energy balance condition with food intake exceeding energy expenditure promotes the development of overweight or obesity^1^. Obesity is strongly associated with type-2 diabetes, a major metabolic disorder producing adverse impacts on human health^1^,^2^. Defining the molecular mechanisms underlying energy balance control is critical for developing effective therapies against obesity and its associated metabolic disorders.

The c-Jun N-terminal kinase (JNK), a member of the mitogen-activated protein kinase (MAPK) family, mediates the responses of cells to environmental stresses^3^. At organismal level, JNK plays a role in controlling global energy homeostasis^4^,^5^,^6^,^7^,^8^,^9^,^10^. High-fat diet feeding induced obesity activates JNK in peripheral organs as well as in the central nervous system^4^,^9^,^11^. Importantly, global knockout of JNK isoform-1 (JNK1), a ubiquitously expressed isoform, protects mice from developing diet-induced obesity (DIO)^9^, which supports a role for JNK, mediated in part by JNK1, in promoting high fat diet-induced obesity. The subsequent studies of tissue-specific JNK1 deletion further demonstrate that the brain plays a major role in mediating JNK1 effect on obesity development^4^,^5^. In this regard, the pituitary, an endocrine gland in the brain, and the hypothalamus, a brain region controlling feeding and energy balance^12^, have been identified as the primary sites in mediating the actions of central JNK1^6^,^7^. In the pituitary, JNK1 appears to enhance the negative feedback control of thyroid-stimulating hormone (TSH) production, which constrains thyroid hormones production^6^. The effect on the pituitary-thyroid axis contributes to a suppression of whole body energy expenditure and underlies JNK1-mediated weight gain and development of obesity^5^,^6^. With respect to the roles of JNK in feeding control, there has not been a consensus. One study showed that JNK1 deficient mice have upregulated expressions of orexigenic neuropeptides in the hypothalamus, and these mice display an elevated hyperphagic response during refeeding following fasting^10^. These results suggest JNK1 action in the brain suppresses food intake^10^. In contrast, another study showed that the activation of JNK1 pathway in the hypothalamus enhances orexigenic signaling, and promotes feeding and weight gain^7^.

In addition to JNK1, the neuron-specific isoform of JNK (JNK3) is also implicated in the control of food intake and body weight homeostasis^8^. A recent study reported that JNK3 deletion in specific neuronal populations, partly by enhancing hypothalamic orexigenic neuronal signaling, promotes high-fat diet intake, and consequently increases the sensitivity of mice to high-fat diet-induced obesity^8^. Thus, based on this study, in contrast with the role of JNK1, hypothalamic JNK3 appears to protect against the development of obesity^8^.

The aforementioned studies are all based upon genetic mouse models, and the developmental compensation inherently associated with genetic approach can seriously confound conclusions. However, pharmacological studies employing specific regulators can complement genetic approach and overcome the issue of developmental compensation. In this regard there is a paucity of pharmacological studies addressing the roles for JNK in controlling feeding and energy balance. Furthermore, JNK has emerged as a promising target of drug design for treating obesity and its associated metabolic disorders^13^. Among the extensive effort in exploring the therapeutic intervention targeting JNK activity, compound SP600125 has been the most characterized regulator of JNK activity with an inhibitory effect on JNK activity^14^,^15^. However, the application of SP600125 to modulate JNK activity has been impeded by its low target selectivity^15^,^16^, and the clinical efficacy of SP600125 is limited by its poor aqueous solubility^10^,^11^,^15^. In this report, we employed compound SR3306 and SR11935, novel JNK inhibitors that are selective, water-soluble and brain-penetrant^17^,^18^, to investigate the roles of JNK in the control of feeding and energy balance as well as in the development of obesity. Our study aims to promote future novel drug design employing JNK inhibitors to treat obesity.

## Results

### Systemic administration of pan-JNK inhibitor SR3306 reduced food intake and induced weight loss in lean mice

**Figure 1:**
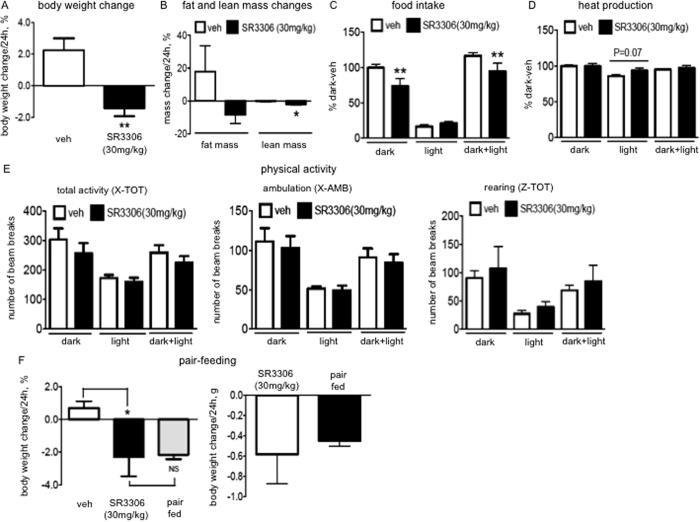
Intraperitoneal (i.p.) administration of SR3306, a brain-penetrant and pan-JNK inhibitor, reduced food intake and induced weight loss. A bolus of i.p. injection of SR3306 (30 mg/kg) or the vehicle (1XPBS) was administered into wild type lean mice before the dark onset. Body weight (**A**) and body composition (**B**) before and 24 hours after the injection were measured (n = 7). The percentage of the change was presented. *P < 0.05, **P < 0.005. (**C**–**E**) Indirect calorimetry was performed with CLAMS in the next 18 hours (12 h-dark cycle and 6 h-light cycle) following the injection. (**C**) The data of food intake from two groups were pooled. The baseline food intakes of the two groups are significantly different, and the absolute amount of food intakes was normalized to that during the dark cycle of the vehicle-treated mice (“dark-veh”, 2.7–3.4 g/12 h) that is set at 100% (n = 9–11). **P < 0.005. (**D**) The data of heat productions from two groups of mice were pooled. The baseline heat productions of the two groups are significantly different, and the absolute amount of heat productions was normalized to that during the dark cycle of the vehicle-treated mice (“dark-veh”, 0.4–0.5 kcal/h) that is set at 100% (n = 9–11). (**E**) Locomotor activity. Total activity (the total number of beam breaks along the x-axis of the CLAMS cage), ambulatory movement (the number of consecutive beam breaks along the x-axis of the CLAMS cage) and rearing (the number of beam breaks along the z-axis of the CLAMS cage) were monitored (n = 6–7). (**F**) A group of mice were provided with the amount of food that was consumed by the mice treated with SR3306 (30 mg/kg) (the same group of mice in Suppl Fig. 1). The percentage of body weight change, and the amount of weight change over 24h-period were presented (n = 6–8). *P < 0.05, NS: not significant.

**Figure 2:**
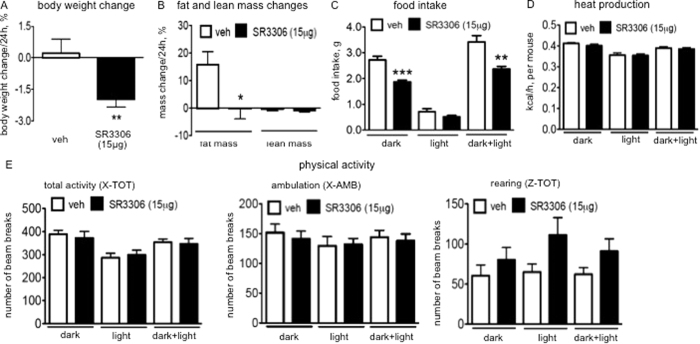
Intracerebroventricular (i.c.v.) administration of SR3306, a brain-penetrant and pan-JNK inhibitor, reduced food intake and induced weight loss. A bolus of i.c.v. injection of SR3306 (15 μg) or the vehicle (1XPBS) was administered into wild type lean mice before the dark onset. Body weight (**A**, n = 8–15) and body composition (**B**, n = 3–5) before and 24 hours after the injection were measured. The percentage of the change was presented. Body weight changes were from two independent experiments, and were pooled. **P < 0.005, *P < 0.05. (**C**–**E**) Indirect calorimetry was performed with CLAMS in the next 18 hours (12 h-dark cycle and 6 h-light cycle) following the injection (n = 5–10). (**C**) Food intake. **P < 0.005, ***P < 0.001. (**D**) Heat production. (**E**) Locomotor activity. Total activity (the total number of beam breaks along the x-axis of the CLAMS cage), ambulatory movement (the number of consecutive beam breaks along the x-axis of the CLAMS cage) and rearing (the number of beam breaks along the z-axis of the CLAMS cage) were monitored.

To investigate the effects of JNK inhibitor SR3306 on feeding and energy balance, we administered the compound into wild type lean mice by intraperitoneal (i.p.) injection. We first performed a dose-response study to determine the effective dosage of altering food intake and body weight. A bolus i.p. injection of SR3306 significantly suppressed overnight food intake and reduced body weight starting at 30 mg/kg, and a dosage of 60 mg/kg further reduced food intake and body weight (SI-Fig. 1). Compound treatment may cause malaise or sickness, which results in feeding inhibition and weight loss. To assess the malaise or sickness-developing potential of SR3306 treatment, we performed conditioned taste aversion (CTA) test^19^ by pairing SR3306 administration with saccharin solution that provides a novel taste^19^. Compound-treated mice will display avoidance to the subsequent intake of saccharin solution if they are made sick by the treatment. We found that i.p. injection of SR3306 with the dose of 30 mg/kg did not affect saccharin intake preference as compared to vehicle (PBS)-treated mice, while the dose of 60 mg/kg elicited a strong avoidance response to saccharin (SI-Fig. 2). The CTA test results indicate that i.p. treatment of SR3306 with the dose of 30 mg/kg elicited anorexia in mice not by malaise or sickness mechanisms, but by homeostatic mechanisms. Thus, we used 30 mg/kg as the dose in i.p. treatment of SR3306 throughout the subsequent studies. SR3306 is highly brain-penetrant, and previous studies demonstrated that systemic administration of the compound exerts a potent inhibitory effect on JNK activity in the substantia nigra area in the brain^18^. As SR3306 exerts its inhibitory effect on JNK activities by competing with ATP (a substrate of JNK activity), thus blocking the kinase action of JNK^18^, we measured the phosphorylation level change in c-Jun, a canonical substrate of JNK, to confirm the action of SR3306. We tested the effect in the hypothalamus in obese mice that have elevated and readily detectable levels of basal JNK activities^4^. As expected, SR3306 treatment reduced the hypothalamic level of phosphorylated c-Jun, (SI Fig. 3A), indicating that the compound inhibits JNK activity in the hypothalamus.

After validating the treatment protocol, we assessed in detail the effects of i.p. administration of SR3306 on food intake and energy balance in lean mice. The mice treated with SR3306 displayed a significant weight loss (Fig. 1A), a strong trend of reduction of fat mass and a significant decrease in lean mass (Fig. 1B). Along with reducing body weight, SR3306 treatment inhibited food intake (Fig. 1C) in the dark cycle when the major portion of feeding activity takes place. Besides food intake, energy expenditure is the other factor determining energy balance and body weight homeostasis. In the dark cycle, SR3306-treated mice did not display changes in heat production (Fig. 1D), indicating energy expenditure was not altered. However, the treated mice displayed an increase in energy expenditure in the following light cycle (Fig. 1D), which was associated with the mild increase, although not significant, in food intake (SR3306: 20% vs. veh: 17%). SR3306 treatment did not affect locomotor activity (Fig. 1E), which indicates that the compound did not suppress feeding by altering physical activity.

Previous studies show that loss of functions of JNK1/2 is associated with elevation of energy expenditure, which underlies the weight loss effects of JNK1/2 inhibition^4^,^5^,^6^. As feeding impacts energy expenditure, we injected SR3306 during the light cycle when mice do not eat significantly. As expected, SR3306 treatment did not affect food intake in the light cycle (Suppl Fig. 4A), and energy expenditure was not altered by SR3306 treatment (Suppl. Fig. 4B). These data clearly show that SR3306-mediated inhibition of JNK activity did not exert a primary effect of stimulating energy expenditure. To confirm that the reduction of food intake is the main cause of weight loss, we performed pair feeding experiment. As expected, the mice that were given the same amount of food as was consumed by the SR3306-treated mice lost the similar body weight as the SR3306-treated ones (Fig. 1F). Taken together, the data show that SR3306 treatment did not cause major changes in energy expenditure, and the SR3306-induced weight loss was mainly accounted for by the feeding inhibition.

### Central administration of SR3306 reduced food intake and induced weight loss in lean mice

Compound SR3306 has a good brain penetration property^18^, and we thus expect SR3306 administered through i.p. route would affect food intake by modulating brain feeding centers. We directly tested the central effects of SR3306 by i.c.v. administration of the compound into lean mice. Similar to the effects by i.p. injection, i.c.v. SR3306 reduced body weight (Fig. 2A) and reduced fat mass (Fig. 2B). Contrasting the peripheral injection, central administration of SR3306 did not affect lean mass, which suggests that the inhibition of JNK activities in the peripheral sites following IP injection produces the effect of reducing lean mass. Indeed, there has been a report indicating that the inhibition of JNK activity could attenuate phosphorylation and activation of p70S6 kinase that mediates aspects of protein synthesis^20^. Along with the loss of body weight, food intake in the dark cycle was suppressed by i.c.v. treatment of SR3306 (Fig. 2C). We further assessed energy expenditure, and demonstrated that heat production (Fig. 2D) was not affected by central injection of SR3306, which is similar to the finding in i.p. injection experiment. Thus, the i.c.v. injection experiment demonstrated that the brain mediates aspects of the effects of systemic JNK inhibition on feeding and energy balance. As in i.p. treatment, the levels of physical activity, indicated by locomotor activity, were not affected by central treatment of SR3306.

Brain JNK1 knockout mice have reduced expression levels of growth hormone, resulting in reduced somatic growth, which contributes to the smaller size of the animals^4^. In our studies, we measured the message level of growth hormone in the pituitary, and did not find significant changes (% veh; veh-treated: 100 ± 16, SR3306-treated: 123 ± 32; P > 0.05).

### SR3306 treatment suppressed food intake during refeeding following fasting

**Figure 3:**
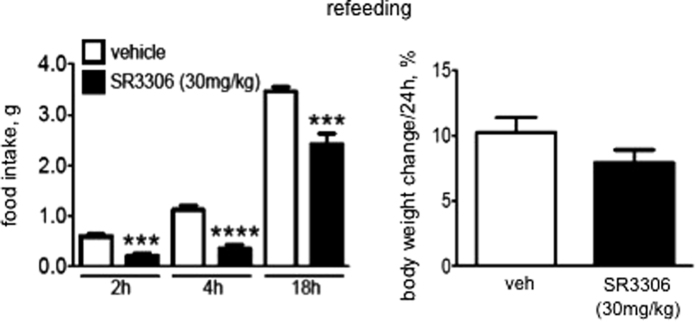
Intraperitoneal (i.p.) administration of SR3306, a brain-penetrant and pan-JNK inhibitor, reduced food intake during refeeding following fasting. The mice were fasted for 18 h. Then, a bolus of i.p. injection of SR3306 (30 mg/kg) or the vehicle (1XPBS) was administered and the food was presented. Food intakes in the next 2, 4 and 18 h, and body weight change following refeeding were monitored (n = 6–7). ***P < 0.001, ****P < 0.0001.

A previous study showed that the JNK-deficient mice display enhanced hyperphagia and weight gain upon refeeding following fasting^10^, suggesting JNK action suppresses feeding. However, a recent report demonstrates that constitutive activation of JNK1 in hypothalamic orexigenic neurons promotes feeding, which leads to increases in adiposity and body weight gain^7^. To tackle the contradiction, we re-assessed the effects of JNK on food intake during refeeding in lean mice treated with SR3306. Here, we showed that the inhibition of JNK by SR3306 suppressed the hyperphagia during refeeding (Fig. 3), in a time course that is consistent with the pharmacokinetic properties of SR3306^18^. In addition, SR3306 treatment did not enhance the weight gain associated with refeeding (Fig. 3). Thus, inhibition of JNK activity attenuated the heightened drive for feeding, which is consistent with an orexigenic effect of JNK activity^7^, but is against the proposed role of JNK1 for restricting energy intake during refeeding^10^.

It should be stressed that these data demonstrated a rapid effect of feeding inhibition (as early as 2 h post-injection) by SR3306 treatment. Similar to the fasted mice, the mice fed *ad. lib.* also displayed a rapid feeding inhibition (as early as 2 h post-injection) by SR3306 treatment (veh-treated: 0.50 ± 0.15 vs. SR3306-treated: 0.21 ± 0.09; P < 0.05).

### SR3306 treatment downregulated SOCS3 level in the hypothalamus in lean mice

The hypothalamus integrates neural, humoral and nutritional cues reflecting energy balance conditions to control feeding and body weight homeostasis^12^. A set of well-known neuropeptides including orexigenic neuropeptide Y (NPY), orexigenic agouti-related peptide (AgRP) and anorexigenic proopiomelanocortin (POMC) mediate aspects of the hypothalamic control of food intake and energy homeostasis^12^. We attempted to explore the molecular mechanisms underlying SR3306-induced changes in feeding and body weight, by analyzing the gene expression levels of these neuropeptides. To exclude the effects of feeding on the expression levels of neuropeptides, we administered SR3306 into fasted animals. We did not find changes in the message levels of NPY, AgRP or POMC (Supp. Fig. 5). Among the various humeral signals received by the hypothalamus, adipocyte-derived hormone leptin is the best studied one, and it is firmly established that leptin plays an essential role in controlling feeding as well as regulating energy balance and body weight^12^. The suppressor of cytokine signaling-3 (SOCS3) is a key inhibitor of leptin intracellular signaling actions^21^, and modulations of SOCS3 level in the hypothalamus impact leptin’s anorectic effect^22^. We demonstrated that SOCS3 expression level was downregulated in response to SR3306 treatment (Supp. Fig. 5), suggesting that leptin’s anorectic effect might be enhanced by JNK inhibition.

### Central administration of a JNK2/3-selective inhibitor SR11935 reduced food intake and induced weight loss in lean mice

**Figure 4:**
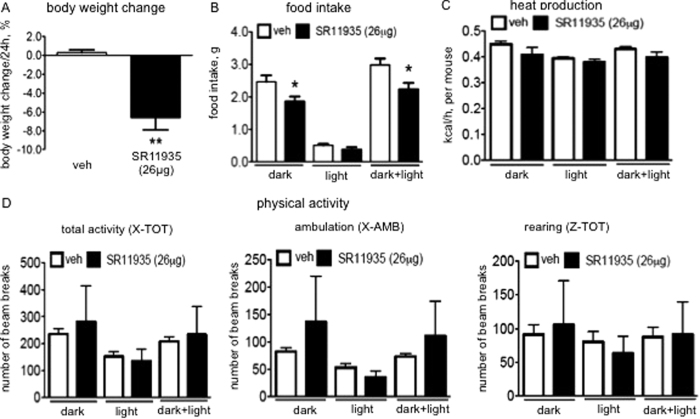
Intracerebroventricular (i.c.v.) administration of SR11935, a JNK2/3 isoform-selective inhibitor, reduced food intake and induced weight loss. A bolus of i.p. injection of SR11935 (26 μg) or the vehicle (1XPBS) was administered into wild type lean mice before the dark onset (n = 5–6). (**A**) Body weight before and 24 hours after the injection were measured. The percentage of the change was presented. **P < 0.005. (**B**–**F**) Indirect calorimetry was performed with CLAMS in the next 18 hours (12 h-dark cycle and 6 h-light cycle) following the injection. (**B**) Food intake. *P < 0.05. (**C**) Heat production. (**D**) Locomotor activity. Total activity (the total number of beam breaks along the x-axis of the CLAMS cage), ambulatory movement (the number of consecutive beam breaks along the x-axis of the CLAMS cage) and rearing (the number of beam breaks along the z-axis of the CLAMS cage) were monitored.

We have demonstrated that pan-JNK inhibition by compound SR3306, induced feeding inhibition and reductions of adiposity and body weight. Based on our current findings and other genetic mouse studies^4^,^5^,^6^,^7^,^9^,^10^, JNK1 and JNK2 appear to play roles in JNK-mediated controls of feeding and energy balance. In addition, JNK3, the isoform that is primarily expressed in the CNS, is also involved in the control of feeding and the regulation of energy homeostasis^8^. To elucidate this role for JNK3, we employed compound SR11935, a novel and highly selective inhibitor of JNK2/3 isoform with a good brain penetration properties^23^. Similar to SR3306, SR11935 inhibits JNK2/3 activity by interfering with ATP binding to JNK’s, and we tested the effect of SR11935 on JNK activity by monitoring the phosphorylation level change in c-Jun. We demonstrated that i.p. injection of SR11935 with the dose adjusted to the equivalent potency of SR3306^23^ induced a decrease in phospho-c-Jun in the hypothalamus (Suppl Fig. 3B), indicating the compound effectively enters the brain to suppress central JNK activity. Although we could not directly confirm an isoform-specific effect in cells in the current studies, our previous study employing purified individual JNK isoforms unequivocally demonstrated that SR11935 has an isoform selectivity of greater than 50-fold for JNK2/3 over JNK1^23^. We then explored the effects of SR11935 on feeding and body weight by intraperitoneally (i.p.) administering the compound. With the equivalent dose, an i.p. bolus injection of SR11935 reduced body weight (Suppl Fig. 6A) and food intake (Suppl Fig. 6B). Moreover, central delivery of SR11935 induced a trend of weight loss (Suppl Fig. 6C) and a significant reduction of fat mass (Suppl Fig. 6D). Surprisingly, along with the decreases in body weight and fat mass, neither food intake (Suppl Fig. 6E) nor energy expenditure (Suppl Fig. 6F) was altered. The lack of changes may reflect the limit of the detection sensitivity of the currently employed methodology. To reveal any potential anorectic effect by central SR11935, we doubled the injection dose. We found i.c.v. administration of the compound with the increased dose induced a marked weight loss (Fig. 4A), accompanied by significant reductions of food intake (Fig. 4B). Similar to i.c.v. SR3306, central treatment of SR11935 did not alter energy expenditure (Fig. 4C). Physical activity was also not affected by SR11935 treatment (Fig. 4D).

### SR3306 treatment prevented body weight gain in response to high-fat diet feeding

**Figure 5:**
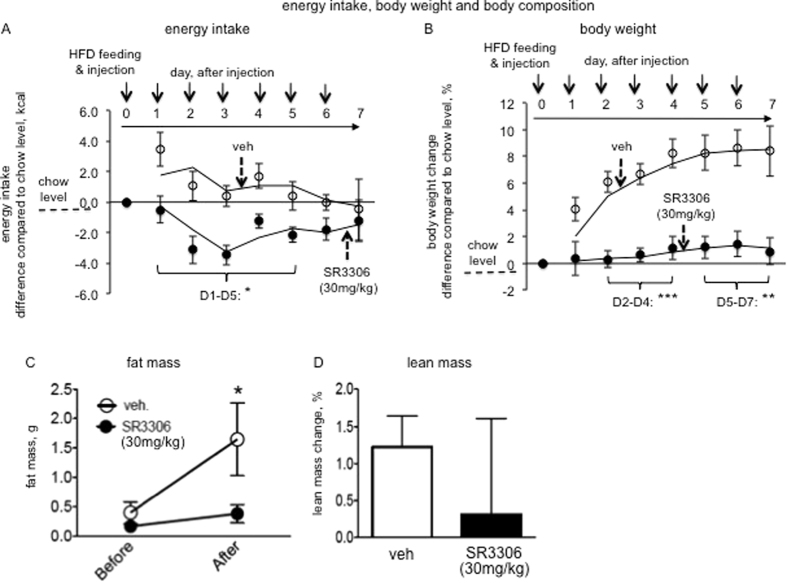
Intraperitoneal (i.p.) administration of SR3306, a brain-penetrant and pan-JNK inhibitor, reduced food intake and prevented body weight gain in response to high-fat diet feeding. Lean mice maintained on chow diet received one bolus of i.p. injection of SR3306 (30 mg/kg) or the vehicle (1XPBS), and were switched to high-fat diet (day 0). The injections were administered in the following 7 consecutive days (n = 4–6). Daily food intake and body weight were monitored. The caloric intakes of high-fat diet were compared to the caloric level of chow intake (10 kcal/24 h), and the differences between the caloric intakes of high-fat diet and the chow diet are presented (the chow intake level was set at 0). The percentages of daily body weight change as compared to the level of day 0 are presented. Following the 7th injection, body composition was measured. *P < 0.05.

We have demonstrated that SR3306-mediated JNK inhibition reduced adiposity and body weight in lean mice fed with regular chow diet. We next investigated the effects of SR3306 on food intake and body weight following high-fat diet (HFD) feeding. Immediately following switching from chow diet to HFD, we administered 7 consecutive daily i.p. injections to the lean mice. Consistent with previous studies^24^, the energy intake of vehicle-treated mice during the first two days (48 h) following HFD feeding were above the chow-fed level (Fig. 5A). In contrast, SR3306-treated animals had a lower level of energy intake than the vehicle-treated mice (Fig. 5A). Starting from the 3rd day, the energy intake of the vehicle-treated mice returned to chow level, while the level of SR3306-treated mice remained significant lower until the 5th day (Fig. 5A). The vehicle-treated animals rapidly gained weight upon HFD feeding, while SR3306 treatment completely abrogated the weight gain during the entire monitoring period (Fig. 5B). Fat masses after the injections were compared to those before the injection, and we found SR3306 treatment significantly reduced fat mass increase following high-fat diet feeding (Fig. 5C). Lean masses were not significantly altered after the injections as compared to the levels before the injection (Fig. 5D).

### SR3306 treatment reduced food intake and obesity, and enhanced leptin’s anorectic effect in diet-induced obese mice

**Figure 6:**
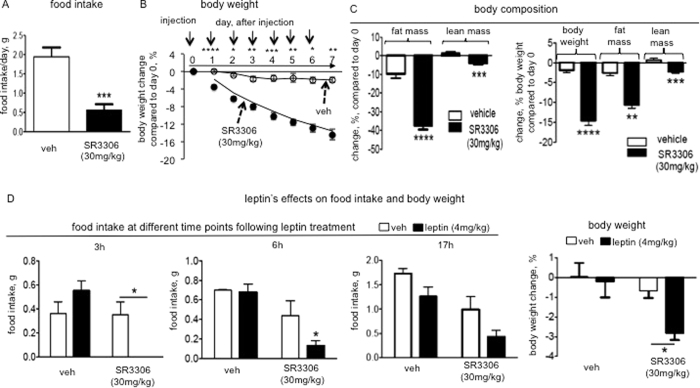
Intraperitoneal (i.p.) administration of SR3306, a brain-penetrant and pan-JNK inhibitor, reduced food intake and body weight, and enhanced leptin-induced anorectic effect in high-fat diet-induced obese (DIO) mice. (**A**–**C**) One bolus of i.p. injection of SR3306 (30 mg/kg) or the vehicle (1XPBS) was administered 2 hours before dark onset per day for 7 consecutive days into diet-induced obese (DIO) mice that are maintained on high-fat diet (n = 6–8). Daily food intake and body weight were monitored. (**A**) The levels of the average daily food intake during the injection period are shown, and (**B**) the percentages of daily body weight change as compared to the level of day 0 are presented. *P < 0.05, **P < 0.005, ***P < 0.001, ****P < 0.0001. (**C**) Following the 7th injection, fat mass and lean mass were measured, and the percent of change, as compared to the levels before the 1st injection is presented. In parallel, the change normalized to the body weight before injection is presented. **P < 0.005, ***P < 0.001, ****P < 0.0001. (**D**) At 1 hour following the last injection, the SR3306-treated and the vehicle-treated mice received a bolus injection of leptin (4 mg/kg) (n = 9) or the vehicle (1XPBS) (n = 5). Food intakes at 3 hours, 6 hours and 17 hours after leptin (or the vehicle) injection and 24-hour body weight change were measured. *P ≤ 0.05.

High-fat diet-induced obese (DIO) mouse is a particularly good model that closely mimics the metabolic derangements in human obesity^25^. We tested the effects of SR3306 treatment on feeding and body weight in DIO mice. We administered the compound intraperitoneally for 7 consecutive days into 22-week old DIO mice that have been maintained on high-fat diet. Along with the injections, the average daily high-fat diet intake (Fig. 6A) as well as the body weight (Fig. 6B) were markedly reduced by SR3306 treatment. Both fat mass and lean mass were reduced by SR3306 treatment, as compared to vehicle treatment (Fig. 6C). When the mass changes were normalized to body weight, we found that the fat mass loss constituted the major portion of weight loss (Fig. 6C, right panel). In these DIO mice, the vehicle-treated ones exhibited reductions of both body weight and fat mass, as compared to the pre-injection levels (Fig. 6B and C). This may be caused by the stress associated with injection procedure and the increased sensitivity of DIO mice to stress^25^.

The resistance to the anorectic action of leptin is a hallmark of diet-induced obesity in rodents^26^. Development of leptin resistance exacerbates DIO, while enhancement of leptin sensitivity protects against DIO^27^. Because the DIO mice are leptin resistant, the enhancement of leptin sensitivity in these mice can be more easily revealed than in lean mice that are already sensitive to leptin’s anorectic effect. We thus tested the sensitivity to leptin’s action of suppressing feeding in the DIO mice that were treated with JNK inhibitor. In the vehicle-treated animals, i.p. injection of leptin failed to reduce food intake (Fig. 6D), demonstrating the presence of leptin resistance in DIO mice. Importantly, in SR3306-treated mice, exogenous leptin markedly suppressed feeding (Fig. 6D). In parallel, the weight-lowering effect of leptin was also restored by SR3306-treatment (Fig. 6D). Together, these data demonstrate that JNK inhibition sensitizes leptin’s effects of inhibiting food intake and lowering body weight.

### SR3306 treatment enhanced leptin-induced STAT3 activation in the hypothalamus of DIO mice

**Figure 7:**
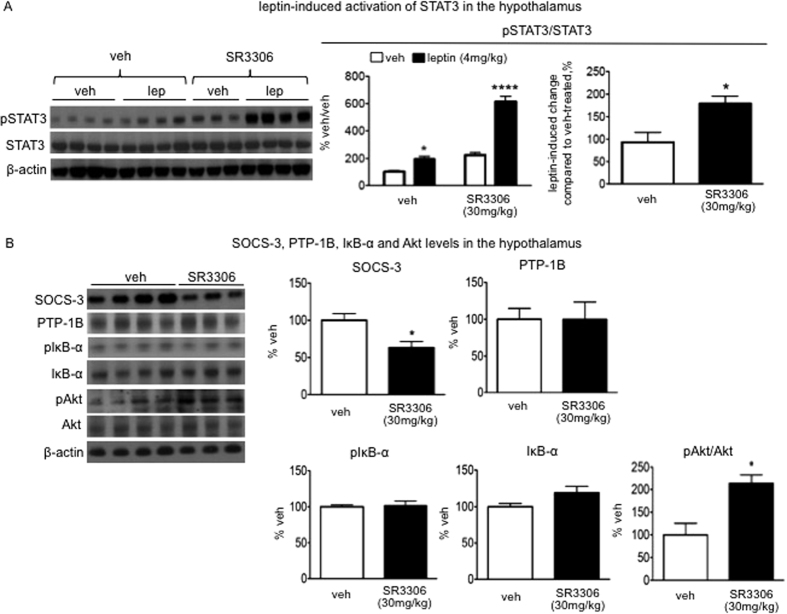
Intraperitoneal (i.p.) administration of SR3306, a brain-penetrant and pan-JNK inhibitor, enhanced leptin-induced activation of STAT3 in the hypothalamus. One bolus of i.p. injection of SR3306 (30 mg/kg) or the vehicle (1XPBS) was administered 2 hours before dark onset per day for seven consecutive days into diet-induced obese (DIO) mice. (**A**) At 1 hour following the last injection, the SR3306-treated and the vehicle-treated mice received a bolus injection of leptin (4 mg/kg) or the vehicle (1XPBS). The animals were sacrificed 30 minutes following leptin (or its vehicle) injection. The mediobasal hypothalamic area encompassing the arcuate nucleus was dissected. The levels of phospho-STAT3 (Tyr705) and STAT3 were measured by Western blotting (n = 3–4). The intensity of phospho-STAT3 (Tyr705) (pSTAT3) was normalized to that of STAT3, and the ratio, pSTAT3/STAT3, was presented. β-actin was used as the loading control. The fold of the change in pSTAT3/STAT3 induced by leptin treatment was compared between the SR3306-treated group and the vehicle-treated group. *P < 0.05, ****P < 0.0001; SR3306/veh. vs. veh./veh.: P < 0.005. (**B**) At 90 minutes following the last injection of SR3306 (or the vehicle), the animals were sacrificed, and the protein levels of SOCS-3, PTP-1B, phospho-IκB-α (Ser32), IκB-α, phospho-Akt (Ser473) and Akt in the mediobasal hypothalamus were measured by Western blotting (n = 3–4). β-actin was used as the loading control. *P < 0.05.

We have shown that JNK inhibition by SR3306 enhanced whole body response to leptin. We next explored the cellular mechanisms underlying the observed sensitization of leptin’s anorectic effects. Signal transducer and activator of transcription-3 (STAT3) is phosphorylated upon leptin stimulation, and it is a canonical mediator in leptin’s intracellular signaling pathways^28^. The level of phospho-STAT3 (pSTAT3) is a marker indicating leptin’s intracellular signaling actions^27^. In our study, SR3306 treatment markedly enhanced the induction of pSTAT3 by leptin, as compared to the vehicle treatment (Fig. 7A), demonstrating an enhanced leptin’s signaling actions by JNK inhibition. We further measured the level of SOCS3, a core inhibitor of leptin-induced STAT3 phosphorylation^21^. In line with the enhancement of STAT3 phosphorylation, SOCS3 level was downregulated by SR3306 treatment (Fig. 7B). In addition to SOCS3, protein tyrosine phosphatase-1B (PTP1B) is another well-known regulator inhibiting leptin signal transduction pathway^29^. Unlike SOCS3, the protein level of PTP1B was not affected by SR3306 treatment (Fig. 7B). In the hypothalamus, activation of the inhibitor of kappa B kinase (IKK) pathway upregulates SOCS3 expression, which plays a key role in the development of high-fat diet-induced leptin resistance^30^. Furthermore, the IKK cascade is linked to JNK actions^31^. We therefore assessed whether the observed change in SOCS3 level would be related to a potential change in IKK activity. We assessed the action of IKK by monitoring the phosphorylation level of inhibitor kappa B-α (IκBα) that is directly phosphorylated by IKK and being subsequently degraded by proteasome^31^. SR3306 treatment did not alter the level of either phospho-IκBα or total IκBα, indicating the IKK activity was not affected by SR3306 treatment. In addition to STAT3 pathway, the signal transduction through insulin receptor substrate-2 (IRS-2) and phosphatidylinositol-3 kinase (PI-3K) also plays an important role in mediating leptin’s anorectic action^32^. Concomitant with the downregulation of SOCS3 expression, the level of phospho-Akt, a marker of PI-3K activation^33^, was increased by SR3306 treatment (Fig. 7B).

## Discussion

In this report, we demonstrate that pharmacological pan-JNK inhibition induced anorectic effect and weight loss in chow-fed lean mice, lean mice upon switching to high-fat diet and DIO mice maintained on high-fat diet. Our data further show that the brain mediates aspects of the anorectic effect of SR3306-induced JNK inhibition. Our findings are in line with the genetic mouse study in which constitutive activation of JNK1 in the hypothalamus induces hyperphagia and increases in adiposity and body weight^7^. The molecular mechanisms underlying the feeding inhibition by SR3306-mediated JNK inhibition may involve the enhancement of leptin’s effect of suppressing feeding. SOCS3, a negative regulator of leptin’s intracellular signaling actions, appeared to mediate the effect of SR3306-induced sensitization of leptin’s anorectic signaling pathway. In both the lean and the obese mice, the expression levels of SOCS3 in the hypothalamus, a key brain site mediating leptin’s effects on feeding and energy balance, were downregulated by SR3306 treatment. Indeed, in the promoter region of SOCS3, the response element binding AP-1, a canonical downstream target of JNK activity^34^, has been identified^35^. Furthermore, it has been shown that the enhancement of SOCS3 expression requires JNK activation^35^. SOCS3 binds to the leptin receptor and suppresses the phosphorylation of STAT3 that is a crucial mediator of leptin’s control of feeding^21^,^36^. We demonstrated that SR3306 treatment enhanced the leptin-induced STAT3 phosphorylation in the hypothalamus, and markedly augmented the inhibitory effect of leptin on feeding. Thus, our data suggest that JNK activity promotes the expression of SOCS3 with a consequent attenuation of leptin-induced STAT3 activation, which diminishes leptin’s anorectic and weigh-lowering effect. Consistent with this model, it has been shown that constitutive activation of JNK1 in the hypothalamus results in hypothalamic cellular leptin resistance as well as systemic leptin resistance^7^. Thus, inhibition of JNK activity (by SR3306) can curtail leptin resistance, at least in part by modulating SOCS3 expression and STAT3 signaling, which enhances leptin’s anorectic and weight-lower effects.

Although it may not play a causal role in DIO, leptin resistance can exacerbate the development of obesity^27^. The enhancement of leptin’s anorectic and weight-lowering effects is considered as a therapeutic strategy in treating high-fat diet-induced obesity^27^. In line with this notion, our study showed that pharmacological inhibition of JNK activity can restore the reduced leptin sensitivity at both cellular and whole-body level, which is expected to contribute to the observed weight loss. Similar to the current study, metformin treatment that augments leptin-induced phosphorylation of STAT3 in the hypothalamus, ameliorates whole-body leptin resistance and causes hypophagia and weight loss in high-fat diet fed obese rats^37^.

Our study provided further evidence supporting a key role of neuronal SOCS3 in diet-induced leptin resistance. Consistent with our findings, neuron-specific deletion of SOCS3 elevates leptin sensitivity and confers resistance to high-fat diet-induced obesity^38^. In addition to the STAT3 pathway, PI-3K pathway also plays a key role in mediating leptin’s effect on feeding and body weight, and the attenuation of PI-3K signaling underlies diet-induced leptin resistance^32^,^39^. Furthermore, SOCS3 is known to act as a negative regulator of PI-3K signaling pathway^40^. Along with downregulating SOCS3 level, SR3306 treatment increased PI-3K signal action in DIO mice. Thus, SR3306-mediated JNK inhibition enhances STAT3 as well as PI-3K signaling pathways, contributing to the amelioration of leptin resistance and obesity in the DIO mice.

The molecular effectors involved in SR3306-induced anorectic effect are unclear. The expression levels of hypothalamic neuropeptides controlling feeding were not altered by SR3306 treatment. These results were not entirely unexpected. STAT3 as a classical transcription factor directly regulates the expression of hypothalamic neuropeptides including AgRP and POMC^36^. However, neuronal specific deletion of STAT3 in either AgRP or POMC neuron does not display significant changes in the message level of AgRP or POMC^41^,^42^. The lack of changes in neuropeptide levels does not diminish the roles of STAT3 in SR3306-induced feeding inhibition. First, STAT3 may impact genes other than these neuropeptides to mediate SR3306’s effect on feeding. Indeed, recent evidence demonstrates that a significant portion of hypothalamic leptin responsive neurons do not express AgRP or POMC^43^. Second, although STAT3 has been well studied as a transcription factor, a small pool of STAT3 was found to localize in mitochondria and regulates mitochondrial respiration independent of its transcriptional activity^44^. Mitochondrial respiration impacts cellular metabolism, and intermediary metabolic processes in the hypothalamus play important roles in controlling feeding and body weight homeostasis^45^. Thus, SR3306-mediated JNK inhibition might modulate mitochondrial STAT3-dependent regulation of cellular energy metabolism, presumably in the hypothalamus, to suppress feeding and reduce body weight. Interestingly, there has been some evidence, arguably suggesting that JNK activity may influence STAT3’s mitochondrial function^46^.

We demonstrated that the SR3306 induced feeding inhibition as early as 2 h after the treatment. Any mechanism involving gene expression, such as the aforementioned enhancements of STAT3/PI-3K pathways (as a result of the downregulation of SOCS3 levels), cannot account for this rapid effect. An acute alteration of neuronal activity affecting neurotransmitter release is expected to be involved in triggering the rapid feeding response. In addition to the well-recognized nuclear function, JNK can translocate to mitochondria to phosphorylate mitochondrial targets^18^,^47^. Thus, SR3306 treatment, by potentially inhibiting mitochondrial JNK, might result in alteration of mitochondrial signaling in the brain, which might induce acute effects on neuronal activity triggering the rapid feeding response. Another potential mechanism is through interference with endocannabinoid system. The brain levels of endocannabinoid are elevated by fasting, and exogenous cannabinoid administration rapidly stimulates feeding (≤1 h)^48^. Interestingly, the signaling through CB1 cannabinoid receptor involves JNK activation^49^. Thus, SR3306-mediated JNK inhibition may have disrupted brain endocannabinoid signaling actions, and consequently blocked the stimulation of feeding by endocannabinoid. Last, it should be mentioned that some extrahypothalamic brain areas are also involved in controlling feeding behavior, so that SR3306-mediated JNK inhibition in these areas may also impact food intake.

Our data are based on pharmacological approach, and have several discrepancies with the findings from the genetic mouse studies. First, SR3306-mediated JNK inhibition produced evident suppression of food intake in both lean and obese mice, while the mice with brain deletion of JNK1 do not display appreciable anorectic effect^4^,^5^. The exact cause of this discrepancy is unclear, but developmental compensation inherent to genetic mouse models can abrogate the potential changes in feeding behavior. In this regard, a good example is that the adult mice with hypothalamic specific neuronal deletions of leptin receptor display increased weight and adiposity, without concomitant changes in food intake, although leptin has a firmly established role in suppressing feeding^50^. Second, our studies clearly show that, at least in lean animals, suppression of JNK activity in the brain does not stimulate energy expenditure, while previous genetic studies suggest the elevation of energy expenditure plays a key role in JNK1-deficiency-mediated weight loss^4^,^5^,^6^. To this end, one study^4^ demonstrated a marked increase in insulin sensitivity in JNK knockout mice, that is concurrent with the increase in energy expenditure. Insulin can act in the brain to stimulate energy expenditure, and there is indeed evidence suggesting that the elevation of energy expenditure can be secondary to the sensitization of insulin signaling^51^. Therefore, the observed increase in energy expenditure in this study may be caused by the enhancement of insulin sensitivity, rather than being a primary outcome of JNK inhibition. Third, in most knockout studies^5^,^6^, the heat production values from the JNK knockout mice are normalized to their body weights that are reduced. This normalization has been repeatedly challenged^52^, and thus the actual changes in energy expenditure of the JNK knockout mice these studies remain unclear. Fourth, our finding that SR11935, a JNK2/3 isoform-selective inhibitor, induced feeding inhibition are inconsistent with the recent report that JNK3 deletion in AgRP neurons or leptin receptor-expressing neurons results in hyperphagia^8^. Feeding behavior is controlled by a neuronal network consisting of different types of neurons. The final readout of phenotype is determined by the integrated actions of all the neurons in the entire network. As SR11935 treatment is expected to target JNK3 in the entire network, we hypothesize that the inhibition of JNK3 activity in the neurons other than the AgRP neurons or the leptin-responsive neurons suppresses feeding. This potential inhibitory effect on feeding would dominate over the orexigenic effects resulting from JNK3 inhibition in the AgRP and the leptin-responsive neurons, which leads to the net outcome of feeding suppression. Taken together with the results from SR3306 studies, our data suggest that the three JNK isoforms, at the whole body level, play redundant roles in promoting feeding.

The data presented herein suggest that JNK activities play key roles in the controls of feeding and energy balance. Pharmacological inhibition of JNK activity can suppress feeding and reduce body weight. In particular, inhibition of JNK activity appears to be effective in preventing or diminishing the development of obesity and ameliorating leptin resistance under high-fat diet feeding condition. The novel JNK inhibitors that we studied here have a great therapeutic potential for treating obesity.

## Methods

### Animals

Mouse experiments were approved by the Institutional Animal Care and Use Committees (IACUC) of the Scripps Research Institute (Jupiter, Florida). All animal experimental procedures were performed in accordance with the guidelines and regulations of animal research set by the Scripps IACUC (Jupiter, Florida). Male, lean or DIO C57BL/6 mice were purchased from the Jackson Laboratory (Bar Harbor, ME). The mice were housed at a constant temperature of 23 °C with food and water provided ad libitum under a 12-hour light, 12-hour dark cycle. The lean mice were maintained on chow diets. The DIO mice were maintained on high fat diet (60% kcal fat) (Research Diets, New Brunswick, NJ). The mice were monitored daily after shipment until body weights became stabilized. The mice subjected to experimental procedures were 22-week old.

### Compounds

SR3306, a pan-JNK inhibitor, and SR11935, a JNK 2/3-selective inhibitor, were synthesized and characterized as described before^17^,^18^,^23^. Leptin (mouse) was from the National Hormone and Peptide Program (NHPP). SR-3306 was dissolved in either hydroxypropyl methylcellulose or 1X phosphate buffered saline (1X PBS). SR11935 and leptin were dissolved in 1XPBS. The compounds were administered by either intraperitoneal (i.p.) injection or intracerebroventricular (i.c.v.) injection with the coordinates of 0.6 mm caudal to bregma, 1.2 mm lateral to midline and 2.2 mm below skull surface. The dosages used in I.C.V. injection were tested via I.P. injection route, and there were no effects on feeding or body weight (data not shown). This demonstrated that the primary cause of the effects on feeding and body weight by central treatment of JNK inhibitors can be fully accounted for by the inhibition of central JNK activities.

### Body composition

The mice were weighed to determine body weight (BW). Fat mass (FM) and total body water mass (TBW) were determined using a NMR spectroscopy analyzer (Bruker Minispec). Lean mass (LM) was calculated as: LM = BW-FM-TBW.

### Food intake measurement

In some experiments, food intake was monitored by BioDAQ system from Research Diets (New Brunswick NJ) according to manufacturer’s instructions. In the other experiments, food intake was monitored concomitant with metabolic parameters measurement by Comprehensive Laboratory Animal Monitoring System (CLAMS).

### Whole body metabolic assessment

Oxygen consumption (VO2), carbon dioxide production (VCO_2_), and locomotor activity were measured by use of CLAMS (Columbus Instruments, Columbus, OH). Heat production (kcal/h/mouse) was calculated using the formula:


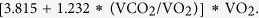


### Conditioned taste aversion (CTA) test

The mice were trained to scheduled, daily, 2-hour water access during the light for 2 weeks. On the first day of the CTA test, the trained mice were given a novel 0.15% saccharin solution to drink for the first 50 minutes, and were then given an i.p. injection of SR-3306 (30 mg/kg or 60 mg/kg) or the vehicle. The injected mice were then provided water for the remaining 70 min. The next day, the mice were allowed to choose between water and 0.15% saccharin for 50 min. Fluid consumption was calculated.

### Real time PCR

The extraction of the total RNA was performed by use of the RNeasy Mini Kit from Qiagen (Valencia, CA). cDNA was synthesized using the high capacity cDNA reverse transcription kit from Applied Biosystems (Carlsbad, CA). PCR was conducted using a 7900 Fast Real-time PCR system, following the instructions from the TaqMan Gene Expression Assays (Applied Biosystems). The message levels of Gapdh were used as the loading control, and it was verified that there were no significant changes in the levels of Gapdh among groups (data not shown).

### Western blotting

Standard immunoblotting procedures were performed according to the protocols detailed by Cell Signaling Technology (Danvers, MA) and Invitrogen (Carlsbad, CA), respectively. The antibodies, except the PTP-1B antibody that is from Santa Cruz Biotechnology (Dallas, TX), are from Cell Signaling Technology. Densitometry was performed with the Scion image software (Frederick, MD).

### Statistical analysis

All data are presented as mean ± SEM unless otherwise noted, Student *t*-test or ANOVA followed by multiple comparison tests (Neuman-Keuls) was used to evaluate the statistical significance. A value of p ≤ 0.05 is defined as statistically significant.

## Additional Information

**How to cite this article**: Gao, S. *et al*. Pharmacological Inhibition of c-Jun N-terminal Kinase Reduces Food Intake and Sensitizes Leptin’s Anorectic Signaling Actions. *Sci. Rep.* **7**, 41795; doi: 10.1038/srep41795 (2017).

**Publisher's note:** Springer Nature remains neutral with regard to jurisdictional claims in published maps and institutional affiliations.

## Supplementary Material

Supplementary Information
