# Supplementary material for: Pharmacological Inhibition of c-Jun N-terminal Kinase Reduces Food Intake and Sensitizes Leptin’s Anorectic Signaling Actions
Source: Sci Rep. 2017 Feb 6;7:41795. doi: 10.1038/srep41795 (PMC5292945; doi:10.1038/srep41795)
Supplement: Supplementary Information [file srep41795-s1.pdf]

**Supplementary Information**

Pharmacological Inhibition of c-Jun N-terminal Kinase Reduces Food Intake and Sensitizes Leptin's Anorectic Signaling Actions

Su Gao*, Shannon Howard, Philip V. LoGrasso

Department of Molecular Therapeutics, Scripps Research Institute, Jupiter, Florida, USA

*Address correspondence to Dr. Su Gao: jhugaosu@yahoo.com

**Suppl Figure 1 Dose-responses of food intake and body weight to i.p. administration of SR3306.** A series of boluses of i.p. injections of SR3306 with different dosages or the vehicle were administered before the dark onset (n=6). Food intake was monitored in the next 18 hours. The body weights right before the injection and 24 hours after the injection were measured, and the percentage of the change was presented. **: 30mg/kg vs veh., P<0.005; ***: 60mg/kg vs veh., P<0.001.

**Suppl Figure 2 Conditioned taste aversion (CTA) test of the single bolus of i.p. injection.** Two dosages of 30 mg/kg and 60 mg/kg of SR3306, by an i.p. bolus injection, were tested. In the CTA test, the fluid intakes at 10 min and 30 min following the presentation of saccharin and water were measured. Total fluid intake, i.e. the summation of saccharin intake and water intake, and the saccharin preference calculated as saccharin intake/total intake were presented (n=5-8). With the dosage of 60 mg/kg, the intake levels are below detection. The data were subject to Kruskal-Wallis Nonparametric test. *: P<0.05.

**Suppl Figure 3 Effects of administration of SR3306 and SR11935 on the phosphorylation level of c-Jun in the hypothalamus.** (A) Some tissue lysates used in Figure 7, i.e. veh (for SR3306)/veh (for leptin) and SR3306/veh (for leptin), were used for immunoblotting of phospho-cJun (Ser 63) (p-cJun) and total c-Jun. At 90 minutes following the last i.p. injection (of SR3306 or its vehicle), the DIO mice were sacrificed. The levels of p-cJun and total c-Jun in the hypothalamus were measured by Western blotting (n=3-4). *: P<0.05. (B) The DIO mice received an i.p. injection of SR11935 (26mg/kg) or its vehicle. At 2h following the injection, the mice were sacrificed. The levels of p-cJun and total c-Jun in the hypothalamus were measured

by Western blotting (n=3). The intensity of p-cJun was normalized to that of total c-Jun, and the ratio, p-cJun/c-Jun, was presented.

**Suppl Figure 4 Effects of administration of SR3306 on food intake and energy expenditure during light cycle.** A bolus of i.p. injection of SR3306 (30mg/kg) or the vehicle (1XPBS) was administered into wild type lean mice in early light cycle. Food intake and heat production were monitored in the next 9 hours during the light cycle (n=5-6).

**Suppl Figure 5 Effects of SR3306 treatment on hypothalamic arcuate nucleus neuropeptide levels.** The mice received a bolus injection of the vehicle or SR3306 (30mg/kg), and were fasted for the next 18 hours. At 18 hours following the injections, the mice were euthanized. The message level in the vehicle-treated group were set as 100%, and used as the control. N=6; *: $P<0.05$. Npy: neuropeptide Y, AgRP: agouti-related peptide, Pomc: proopiomelanocortin, Socs3: suppressor of cytokine signaling-3.

**Suppl Figure 6 Effects of i.p. and i.c.v. administrations of SR11935 on feeding and body weight.** (A and B) A bolus of i.p. injection of SR11935 (26mg/kg) or the vehicle (1XPBS) was administered before the dark onset (n=5-10). (A) Body weight before and 24-hour after the injection were measured. ***: $P<0.001$. (B) Cumulative food intake over the 18h-period after the injection was measured. **: $P<0.005$. (C-F) A bolus of i.c.v. injection of SR11935 (13µg) or the vehicle (1XPBS) was administered before the dark onset (n=8-11). Body weight (C) and body composition (D) before and 24-hour after the injection were measured. The percentage of the change was presented. *: $P\leq0.05$. (E) The data of food intake from two groups were pooled. (F) The data of heat productions from two groups of mice were pooled. The baselines heat production of the two groups are significantly different, and the absolute amount of heat productions was normalized to that during the dark cycle of the vehicle-treated mice (“dark-veh”, 0.4-0.46 kcal/h) that is set at 100%.

Dose-responses of feeding and body weight change by IP administration of SR3306

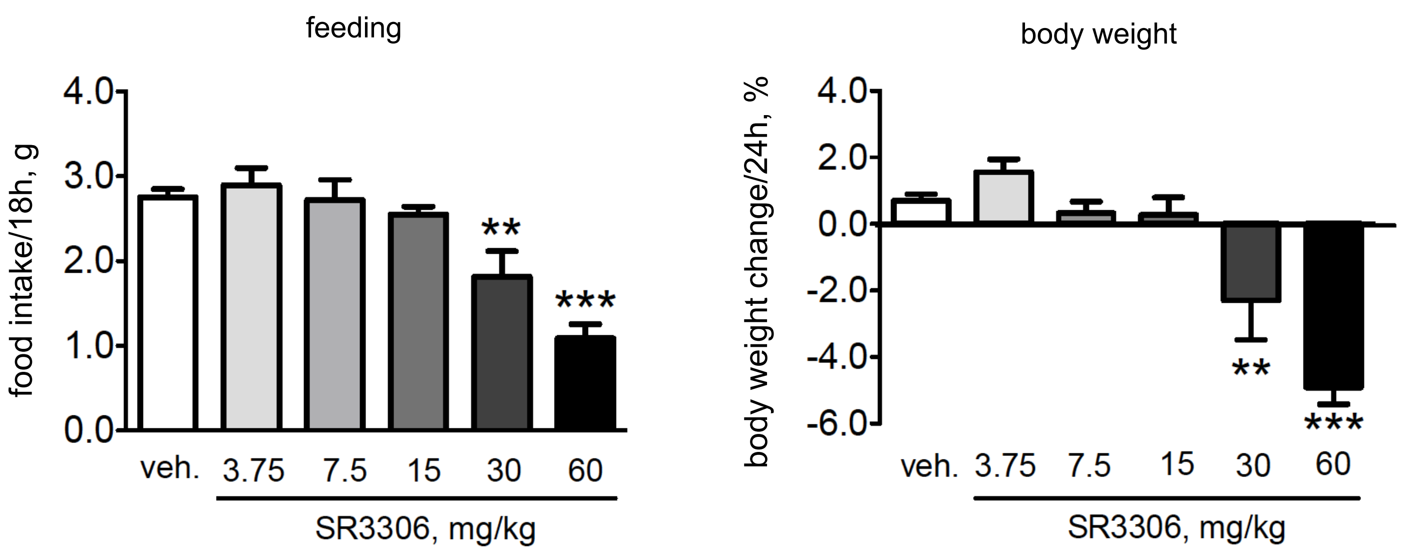


**Suppl. Figure 1**

Conditioned taste aversion test of IP administration of SR3306

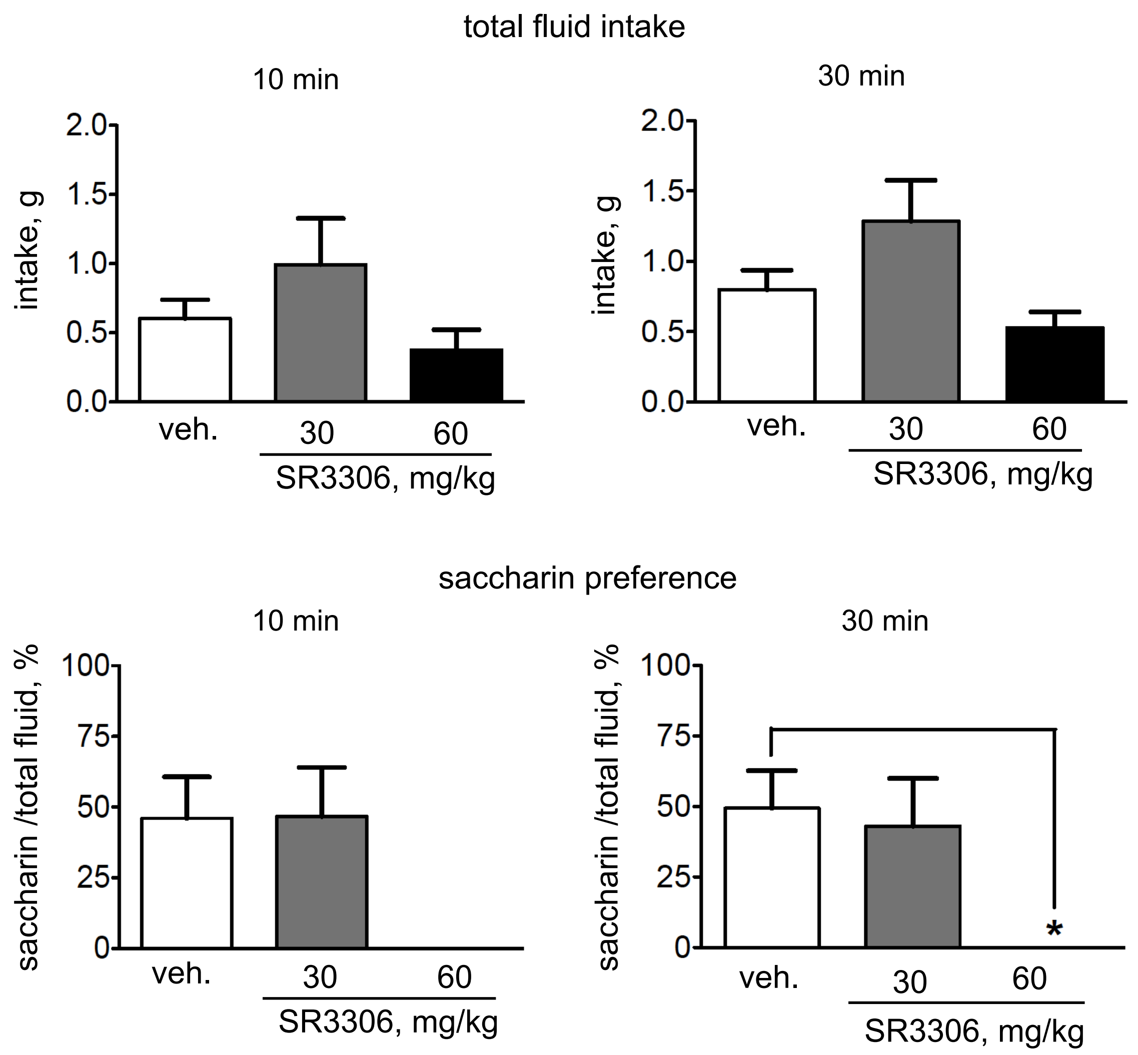


**Suppl. Figure 2**

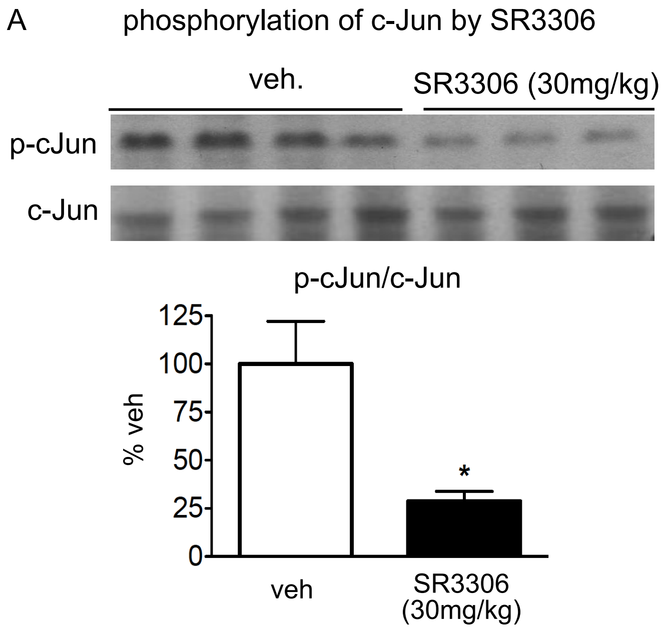


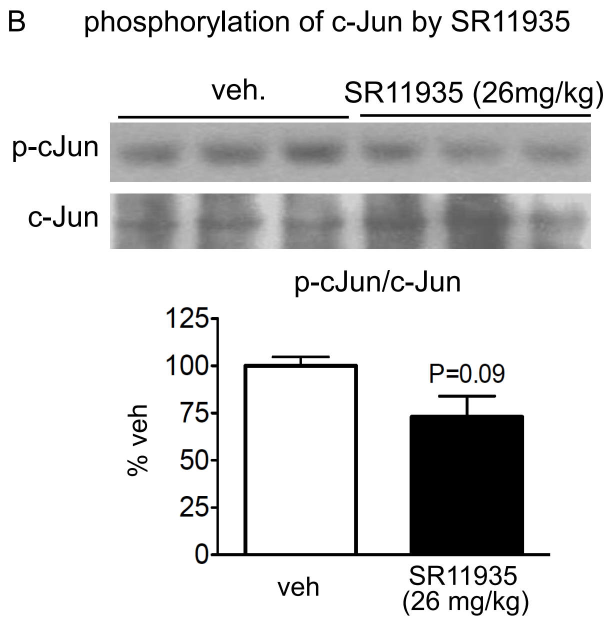


**Suppl. Figure 3**

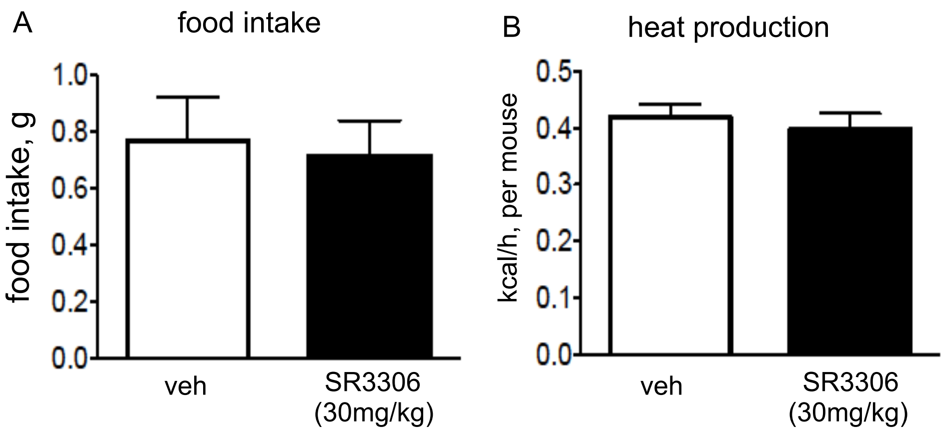


**Suppl. Figure 4**

Hypothalamic arcuate nucleus neuropeptide levels following administration of SR3306

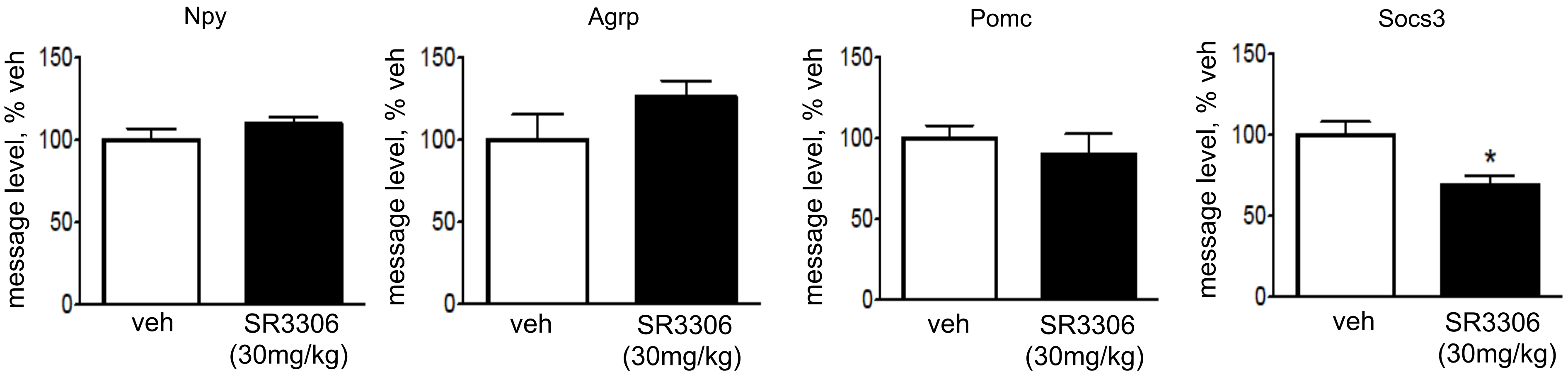


**Suppl. Figure 5**

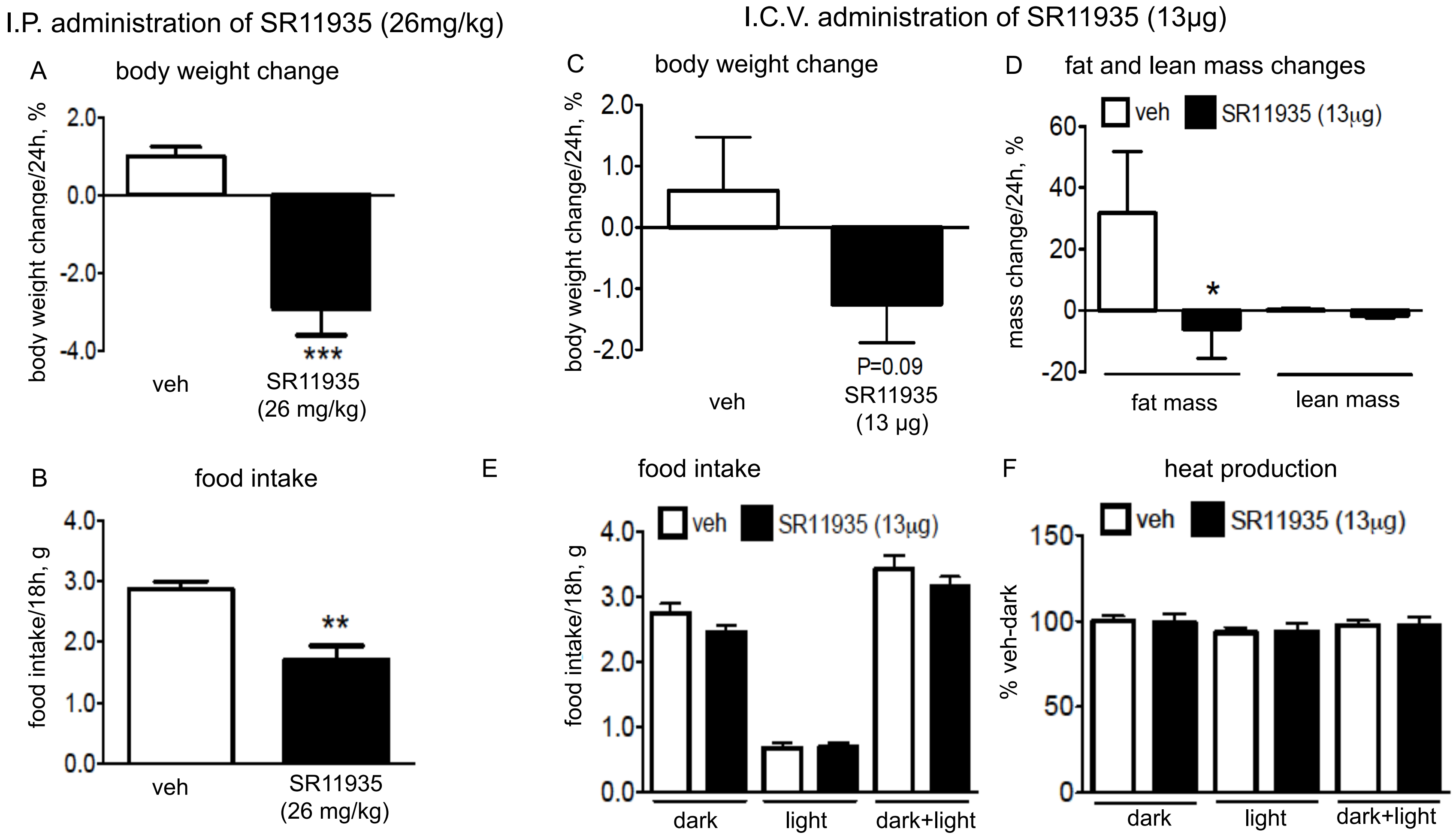


**Suppl. Figure 6**
